# Correction to ‘Mechanisms and Research Progress of Leech in Diabetic Ulcer Fibrosis’

**DOI:** 10.1111/iwj.71024

**Published:** 2026-08-25

Y. Wang , X. Zheng , H. Que , and Z. Yan , “Mechanisms and Research Progress of Leech in Diabetic Ulcer Fibrosis,” International Wound Journal 23, no. 8 (2026): e71013, 10.1111/iwj.71013.42578589 PMC13459645

In the Funding section, the grant number ‘Innovation and Entrepreneurship Training Plan for College Students in Jiangxi Province (S202510412051)’ was incorrect. This should have read: ‘Innovation and Entrepreneurship Training Plan for College Students in Jiangxi Province (S202510412130).’

We apologise for this error.

